# Topologically knotted deubiquitinases exhibit unprecedented mechanostability to withstand the proteolysis by an AAA+ protease

**DOI:** 10.1038/s41598-018-25470-0

**Published:** 2018-05-04

**Authors:** Manoj Kumar Sriramoju, Yen Chen, Yun-Tzai Cloud Lee, Shang-Te Danny Hsu

**Affiliations:** 1 Institute of Biological Chemistry, Academia Sinica, Taipei, 11529 Taiwan; 2 Institute of Biochemical Sciences, National Taiwan University, Taipei, 106 Taiwan

## Abstract

More than one thousand knotted protein structures have been identified so far, but the functional roles of these knots remain elusive. It has been postulated that backbone entanglement may provide additional mechanostability. Here, we employed a bacterial proteasome, ClpXP, to mechanically unfold 5_2_-knotted human ubiquitin C-terminal hydrolase (UCH) paralogs from their C-termini, followed by processive translocation into the proteolytic chamber for degradation. Our results revealed unprecedentedly slow kinetics of ClpXP-mediated proteolysis for the proteasome-associated UCHL5: ten thousand times slower than that of a green fluorescence protein (GFP), which has a comparable size to the UCH domain but much higher chemical and thermal stabilities. The ClpXP-dependent mechanostability positively correlates with the intrinsic unfolding rates of the substrates, spanning over several orders of magnitude for the UCHs. The broad range of mechanostability within the same protein family may be associated with the functional requirements for their differential malleabilities.

## Introduction

Knotted proteins are tantalizing examples of how polypeptide chains can attain intricate folding topologies spontaneously^1–3^. Systematic surveys of the rapidly expanding protein database (PDB) revealed over one thousand knotted protein structures, representing nearly 1% of the total entries^4,5^. These findings indicate that protein knots are not anomalies in the protein universe. In fact, knotted proteins are often enzymes that play key biological functions in all kingdoms of life, such as 3_1_ knotted bacterial RNA methyltransferases^6,7^, a 4_1_ knotted light-sensing phytochrome^8^, 5_2_ knotted ubiquitin C-terminal hydrolases (UCHs)^9,10^ and the most complex 6_1_ knotted dehalogenase^11,12^. Some knotted structural elements are implicated in substrate recognitions, suggesting their functional importance as key structural motifs that are preserved throughout evolution^6,7,9,10^.

While several theoretical models have been proposed to explain how protein knotting may be achieved^13–20^, it remains very challenging to experimentally visualize the knotting events along the folding pathway(s) of a given protein^2,21^. Over the years, a series of systematic experimental analyses on the folding dynamics and kinetics of a broad range of knotted proteins have been reported^22–29^, and a recurrent feature is the presence of highly populated folding intermediates, indicating rugged free energy landscapes associated with the folding knotted proteins. The folding stabilities of knotted proteins have so far been assessed exclusively by chemical denaturation (using urea or guanidium hydrochloride (GdnHCl) as denaturants) and to a lesser extent by thermal denaturation. These experimental findings, however, provided no obvious correlation between the complexity of the knot types and the degree of folding stabilities (Fig. S1). Furthermore, knotted proteins are not necessarily more stable (chemically or thermally) than unknotted proteins of comparable sizes, suggesting that protein knots do not warrant enhanced chemical or thermal stabilities^30^.

We recently observed the exceedingly slow unfolding rate of the proteasome-associated UCHL5 (also known as UCH37), compared to its paralogs, UCHL1 and UCHL3^29^. We postulated that the slow unfolding kinetics of UCHL5 (defined as the slowest intrinsic unfolding rate, ${k}_{u}^{{H}_{2}O}$) might be beneficial for its deubiquitinating activity in a tug-of-war with the proteasome. In other words, UCHL5 may exhibit enhanced mechanostability when subject to mechanical unfolding by AAA+ proteases. Indeed, protein knots have been proposed to afford enhanced folding stability when subject to mechanical unfolding^31^, and theoretical calculations have been applied to model how knotted protein may respond to mechanical unfolding in a simple tubular model^32^ and in a more realistic proteasome-like model system^31,33^, Mechanical unfolding of UCHL1 has been investigated by single molecule optical tweezers experiments^34^. The relaxation (refolding) rates upon the release of mechanical constraints were comparable to the global refolding rate observed by rapid dilution of urea-denatured UCHL1, suggesting that the refolding kinetics of UCHL1 is not significantly biased by the choice of physico-chemical perturbation variables. In order to evaluate the significance of the mechanical stabilities of the knotted UCHs in the context of proteasome-mediated protein degradation, we utilized the archetypical bacterial ClpXP AAA+ (ATPases Associated with diverse cellular Activities) protease system that contains an AAA+ unfoldase component similar to the one in the mammalian proteasome to mechanically unfold target substrates. ClpXP selectively recognizes a specific ssrA tag fused to the C-terminus of a substrate protein to vectorially unfold the substrate via the ATP hydrolysis-dependent mechanical power stroke of the hexameric ClpX AAA+ unfoldase, followed by translocation of the unfolded substrate into the chamber of the proteolytic, tetradecameric ClpP for degradation^35,36^. Single-molecular force spectroscopy has been applied to investigate a variety of ssrA-tagged substrates of different structures and topologies, including GFP. These investigations establish that higher mechanostabilities of ssrA-tagged substrates result in slower ClpXP-mediated proteolysis^37–40^, thus serving as a basis to infer the mechanostabilities of the knotted UCHs based on ClpXP enzyme kinetics.

Our results showed that the ssrA-tagged human UCHs, namely UCHL1, UCHL3, as well as the UCH domains of UCHL5 and BRCA1-associated protein 1 (hereafter denoted as UCHL5 and BAP1 for short), were highly resistant to ClpXP-mediated proteolysis considering that their chemical and thermal stabilities were much lower than that of green fluorescence protein (GFP), which has been the model substrate for ClpXP. Of the four UCHs, UCHL5 exhibited the slowest ClpXP-mediated proteolysis kinetics, on the timescale of many hours instead of a few minutes for GFP. These results suggested that the complex knotted folding topology of UCHs may confer enhanced mechanical stability to withstand the unidirectional unfolding and processive degradation by ClpXP.

## Results

**Figure 1:**
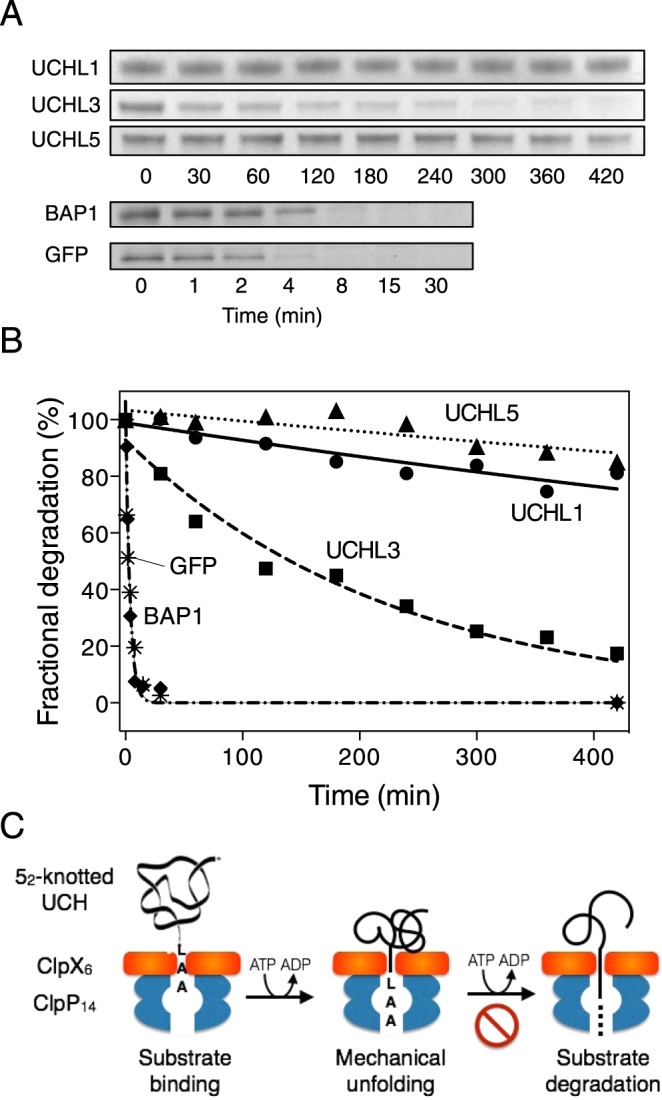
ClpXP-mediated proteolysis of ssrA-tagged UCHs monitored by SDS-PAGE. (**A**) SDS-PAGE images of individual substrates as indicated on the left of each panel. Aliquots were taken at specific time points as indicated below. (**B**) Quantitative image analyses of the results shown in (A) normalized with respect to the initial time point. The results were fit to a single exponential decay function to deduce the apparent life times of individual substrates. The data points represent the mean of three independent degradation assays. (**C**) Schematic presentation of how the knotted UCHs may withstand the mechanical unfolding of ClpXP thereby hindering the subsequent proteolysis.

We initially monitored ClpXP-mediated proteolysis of ssrA-tagged protein substrates by SDS-PAGE analysis. The amounts of intact proteins at various time points of incubation with ClpXP were quantified and fit to a single exponential decay function to estimate the apparent lifetimes of the individual substrates (Fig. 1). While GFP was fully degraded by ClpXP under our experimental condition within 10 minutes with an apparent lifetime of 2.4 minutes, consistent with previously reported findings (Table 1)^41,42^, significant amounts of residual UCHL1 and UCHL5 remained clearly visible in the SDS-PAGE after seven hours of incubation with ClpXP. The apparent lifetimes of ClpXP-mediated proteolysis were 1039 and 1295 minutes for UCHL1 and UCHL5, respectively, indicating their strong resistance against ClpXP-mediated proteolysis. Meanwhile, the corresponding lifetimes for UCHL3 and BAP1 were 155 and 2.7 minutes, respectively, indicating that the mechanical stabilities of proteins with the same folding topology, as assessed by the ClpXP-mediated proteolysis, can vary significantly. This is similar to the observations based on chemical and thermal denaturation. The lack of distinct peptide fragments over the course of ClpXP-mediated proteolysis (judging by the SDS-PAGE analysis that showed only intact UCHs bands with decreasing band intensities) of all ssrA-tagged UCHs indicates that the processivity of ClpXP enzyme activity was retained despite the drastically reduced turnover rates.

**Table 1:** Lifetime of ClpXP-mediated proteolysis derived from SDS-PAGE analysis.

Protein	Lifetime (min)
GFP	2.4 ± 0.4
UCHL1	1039 ± 182
UCHL1_I93M_	391 ± 60
UCHL1_∆11_	37 ± 4
UCHL3	155 ± 20
UCHL5	1295 ± 476
BAP1	2.7 ± 0.4

Considering (i) that the transition concentrations of urea-mediated chemical denaturation, [D]_50%,urea_, of UCHs were less than 3 M whereas GFP did not unfold even at 8 M urea, (ii) that the transition concentrations of GdnHCl -mediated unfolding^20^, [D]_50%,GdnHCl_, were less than 2.5 M whereas that of GFP was 3.01 M (Figs S2, S3 and Table S1), and (iii) that the melting temperatures (*T*_m_) of the UCHs range between 45 and 60 °C, which are at least 20 °C lower than that of GFP (80.1 °C) (Fig. S4 and Table S2), it is remarkable that the least stable of all UCHs studied herein, *i.e*., BAP1 ([D]_50%,GdnHCl_ = 2.04 M and *T*_m_ = 46.9 °C) was mechanically more stable than GFP in the context of ClpXP-mediated proteolysis.

To dissect the contributions of global (chemical and thermal) folding stability and folding topology to the mechanostability of UCHs, we generated two ssrA-tagged UCHL1 variants harboring a Parkinson’s disease (PD)-associated I93M mutation (UCHL1_I93M_)^43,44^, or a naturally occurring truncation of the first eleven residues at the N-terminus (UCHL1_∆11_)^45^. These natural variants of UCHL1 represent two different kinds of perturbations on the folding of UCHL1. On the one hand, the missense I93M mutation destabilizes the folding stability without affecting the overall structure and topology of UCHL1^46^. On the other hand, the N-terminal truncation removes one of the five key projected crossings of the native 5_2_ Gordian knotted folding topology, rendering the truncated UCHL1 unknotted according to previously reported knot detection algorithms^4,5^. Both the I93M mutation and the N-terminal truncation reduced the stability of UCHL1 without global unfolding – the reductions in the [D]_50%,GdnHCl_ value were 0.08 and 0.66 M for UCHL1_I93M_ and UCHL1_∆11_, respectively, and the reduction in thermal stabilities were 3.8 and 9.7 °C, respectively, in Δ*T*_m_ values. The intrinsic unfolding rate of UCHL1_∆11_ (${k}_{u}^{{H}_{2}O}$ = 2.1 × 10^−4^ s^−1^) was two orders of magnitude faster than that of UCHL1 (${k}_{u}^{{H}_{2}O}$ = 7.0 × 10^−6^ s^−1^), which agreed with theoretical prediction that the presence of knot enhances kinetic stability^19^, with a very small *m*-value of unfolding (0.21 kcal mol^−1^ M^−1^) compared to that of UCHL1 (0.97 kcal mol^−1^ M^−1^); in contrast, UCHL1_I93M_ exhibited a three-fold increase in intrinsic unfolding rate relative to UCHL1 (${k}_{u}^{{H}_{2}O}$ = 2.2 × 10^−5^ s^−1^) with a comparable *m*-value of unfolding (1.11 kcal mol^−1^ M^−1^) (Fig. S5 and Table S3). Indeed, when subject to ClpXP-mediated proteolysis, the apparent lifetimes of UCHL1_I93M_ and UCHL1_∆11_ were reduced 2.6- (391 minutes) and 28- (37 minutes) fold, respectively, compared to that of wild-type UCHL1 (Fig. S6), implying that the release of topological constraints at the N-terminal region of UCHL1 has a much more pronounced impact on its mechanostability than perturbation of the hydrophobic core by a single point mutation, and that the cooperativity (*m*-value) may a good indicator of the mechanostability of a ClpXP substrate.

**Figure 2:**
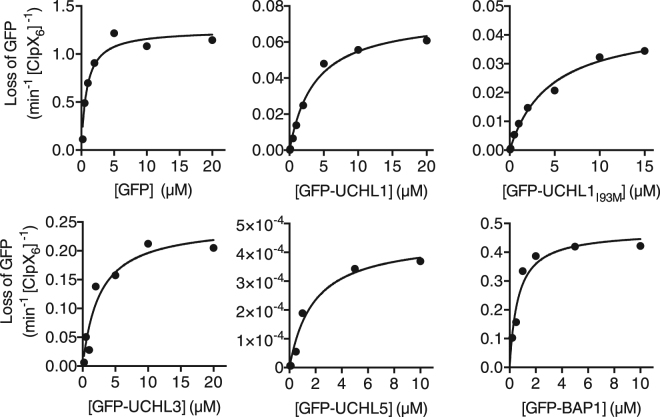
Michaelis-Menten analyses of GFP-CHs. Representative results of triplicates were shown for individual substrates. The initial rates of ClpXP-mediated proteolysis at various GFP-UCHs concentrations (as indicated below the X-axis of each panel) were extracted from fitting the changes in GFP fluorescence within the first 60 seconds.

To quantitatively characterize the mechanostabilities of ssrA-tagged UCHs and the enzyme activity of ClpXP for these substrates, we fused the same GFP construct used for the ClpXP-mediated proteolysis to the N-termini of individual UCHs (hereafter denoted as GFP-UCHs) for Michaelis-Menten analyses of ClpXP-mediated proteolysis by monitoring the reduction of GFP fluorescence as a readout of substrate turnover. As a control, we first established that fluorescence intensity of GFP decayed at the same rate as the density of the intact GFP band separated by SDS-PAGE (Fig. S7). We next showed that the Michaelis-Menten parameters derived from the GFP fluorescence-based analysis under our experimental condition (*V*_max_ = 1.25 min^−1^ [ClpX_6_]^−1^ and *K*_M_ = 0.82 μM; Table 2) were consistent with previously reported results^41^. Finally, by monitoring the in-gel GFP fluorescence and Coomassie blue staining of the same SDS-PAGE gels, we confirmed the processivity and complete turnover of GFP-fused UCHs without distinct fragments, *i.e*., aberrant proteolysis products due to release from the ClpXP, over the course of ClpXP-mediated proteolysis. Having established that the GFP fusion is a good structural reporter of ClpXP-mediated proteolysis, we repeated the same Michaelis-Menten analyses for the GFP-UCHs (Fig. 2). The results showed that while the *K*_M_ values varied between 0.64 and 4.47 μM, which was up to five-fold higher than that of GFP, the *V*_max_ values varied by several orders of magnitude. GFP-UCHL5 exhibited the slowest *V*_max_ value at 4.5 × 10^−4^ min^−1^ [ClpX_6_]^−1^, followed by GFP-UCHL1 and GFP-UCHL1_I93M_ (0.05 and 0.07 min^−1^ [ClpX_6_]^−1^), GFP-UCHL3 (0.25 min^−1^ [ClpX_6_]^−1^), and GFP-BAP1 (0.48 min^−1^ [ClpX_6_]^−1^). These results therefore indicate that the structures of UCHs do not impede substrate (the C-terminal ssrA tag) recognition of ClpXP, but the turnover rates were greatly reduced due to the complex knotted topology. Furthermore, the *V*_max_ values of GFP-fused UCHs are on a comparable timescale as that of the intrinsic unfolding rates of UCHs, implying that global unfolding is the rate-limiting step of ClpXP-mediated proteolysis.

**Table 2:** Michaelis-Menten analysis of GFP-UCHs. The reported mean values and standard deviations were derived from three independent measurements.

Protein	*V*_max_ (min^−1^ [ClpX_6_]^−1^)	*K*_M_ (µM)
GFP	1.25 ± 0.08	0.82 ± 0.20
GFP-UCHL1	0.07 ± 0.005	3.67 ± 0.59
GFP-UCHL1_I93M_	0.05 ± 0.003	4.47 ± 0.79
GFP-UCHL3	0.25 ± 0.03	2.59 ± 1.04
GFP-UCHL5	(4.5 ± 0.4) × 10^−4^	1.85 ± 0.58
GFP-BAP1	0.48 ± 0.04	0.64 ± 0.18

## Discussion

The current study was motivated by our recent observation that the human proteasome-associated UCHL5 exhibits a very slow intrinsic unfolding rate, ${k}_{u}^{{H}_{2}O}$, which is on the timescale of 10^−8^ sec^−1^, leading to our hypothesis that the slow unfolding could be beneficial for executing its biological function in a tug-of-war with the mechanical unfolding of ubiquitinated substrates by the proteasome^29^. To evaluate the mechanostability of UCHL5 and that of its paralogs, we studied the kinetics and enzyme mechanism of ClpXP-mediated proteolysis of ssrA-tagged UCHs. Our current results indicated that the ssrA-tagged UCHL5 and its paralogs were highly resistant to ClpXP-mediated proteolysis. ClpXP is a well-characterized bacterial AAA+ proteasome system that resembles the mechanical unfolding by the AAA+ unfoldase activity of the human proteasome. Recently, the smallest 3_1_ trefoil knotted protein MJ0366 was shown to impair the ClpXP-mediate proteolysis when fused with a GFP at its N-terminus in a linker length-dependent manner^47^. The ssrA-tagged, homodimeric MJ0366 by itself is degraded by ClpXP at a rate of 3 min^−1^, which is comparable to GFP. With an optimized linker length between GFP and MJ0366, the rate of ClpXP-dependent proteolysis is only reduced ten-fold.

**Figure 3:**
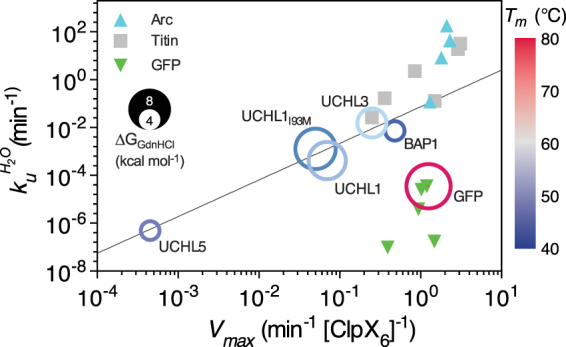
Correlation plots of ClpXP-mediated proteolysis rates, V_max_, with respect to intrinsic unfolding rate ${k}_{u}^{{H}_{2}O}$. The data points of UCHs and GFP are shown in open circles with their radii proportional to the free energy of unfolding ΔG_GdnHCl_ as indicated on the left. Since UCHL1 variants exhibited a three-state equilibrium (Table S1), their radii correspond to the sum of ΔG_N-I_ and ΔG_I-D_. Furthermore, the data points are color-ramped from blue to grey to red according to their melting temperatures, *T*_m_, as indicated on the right. The linear regression of the double-log plot of UCHs data points yields a slope of 1.48 ± 0.18 and a log(${k}_{u}^{{H}_{2}O}$) intercept of −3.10 ± 0.31 when log(V_max_) = 0 (R^2^ = 0.96), shown in solid black line. The previously reported data of Arc (cyan triangles), titin I27 (Titin; grey squares) and GFP (inverted green triangles) are shown for comparison (Table S4).

The relatively fast ClpXP degradation rate was attributed to (i) unknotting of MJ0366 when the N-terminus is too short to tighten the knotted core during the mechanical pulling by ClpXP, and/or (ii) a tightened 3_1_ knot that is sufficiently small to allow direct translocation across the pore of ClpX into the degradation chamber of ClpP while being knotted. In this regard, the much bulkier and monomeric 5_2_ Gordian knotted UCHs (with a diameter of ca. 4 nm that is estimated to be three times larger than a tightened 3_1_ protein knot of MJ0366), which possess higher topological friction than do the 3_1_ knotted proteins^48^ are much better substrates for assessing the effects of protein knots on the ClpXP-dependent proteolysis. To the best of our knowledge, our current results represent the slowest ClpXP-mediated proteolysis kinetics of all protein systems that have been investigated so far, including the well-characterized, highly stable GFP that served as a reference in our current study^37^. Comparison with the previously reported *V*_max_ values of the ClpXP-mediated proteolysis of different model systems revealed a strong correlation between the intrinsic unfolding rates, ${k}_{u}^{{H}_{2}O}$, and the rate of ClpXP-mediated proteolysis, *V*_max_ (Fig. 3), except for GFP, which is an outlier among these protein systems as previously reported^37^. Such a correlation lacks for thermal stability (*T*_m_) and chemical stability, *i.e*., free energy of unfolding derived from equilibrium unfolding by urea or GdnHCl (ΔG_urea/GdnHCl_), indicating that the global thermal and chemical stabilities do not necessarily contribute to the mechanostability of a target protein (Fig. S8).

Despite sharing the same folding topology across the four human UCHs, the kinetics of their ClpXP-mediated proteolysis varied by four orders of magnitude, indicating that the structure and folding topology are not the only contributing factors to their mechanostabilities. It follows that sizeable differences in unfolding kinetics between protein homologs of the same fold are well documented^29,49^. In the case of titin I27 variants harboring different point mutations with a broad range of unfolding kinetics, their mechanostabilities deduced from atomic force microscopy pulling analyses have been shown to correlate well with the efficiency of mitochondria import when a mitochondria-targeting sequence is fused to the N-termini of the titin I27 variants, suggesting a functional role of protein malleability in cellular trafficking and translocation^50^. For example, BAP1 functions as an oncogene suppressor by deubiquitinating important transcription-associated regulatory factors, such as histone H2A and host cell factor 1 (HCF-1)^51^. Nuclear translocation of BAP1 is tightly regulated by its autodeubiquitination^52^, suggesting that BAP1 needs to be shuttled between nucleus and cytosol, and that BAP1 needs to be sufficiently malleable to be transported across the nuclear pore complex. In this regard, BAP1 is positioned at the other end of the spectrum of the mechanostability and unfolding kinetics distributions of UCHs because of its functional requirements for additional malleability. Indeed, BAP1 exhibited the lowest *m*-value associated with kinetic unfolding (0.35 kcal mol^−1^ M^−1^) all four UCHs in contrast to the highest value for UCHL5 (1.88 kcal mol^−1^ M^−1^; Table S3), suggesting that the cooperativity (*m*-value) of chemical denaturation may be a good indicator of the mechanostability of a ClpXP substrate.

## Conclusion

In this study, we have engineered a panel of ssrA-tagged and 5_2_ Gordian knotted UCHs to assess their mechanostabilities in the context of knotted topology using the kinetics of ClpXP-mediated proteolysis as an indirect readout. Our results revealed unprecedentedly slow kinetics of ClpXP-mediated proteolysis of the proteasome-associated UCHL5 that is four orders of magnitude slower than that of GFP. All knotted UCHs are chemically and thermally less stable than GFP, but even the least stable BAP1 is mechanically more stable than GFP in the context of ClpXP-mediated proteolysis. Compilation of previously reported data with our current findings revealed a strong correlation between the intrinsic unfolding rates and the kinetics of ClpXP-mediated proteolysis; GFP is the only outlier that appears to be more susceptible to ClpXP-mediated proteolysis despite its very high chemical and thermal stability (Fig. 3). The significance of the protein knots in the mechanostability of UCHL1 was indirectly assessed by N-terminal truncation (UCHL1_∆11_) that essentially unknotted the 5_2_ knotted topology, resulting in a profound acceleration of the ClpXP-mediated proteolysis. Because the unknotted topology of UCHL1_∆11_ unfolds orders of magnitude faster than the knotted ones, it is more likely to translocate through the ClpXP axial pore in a more unfolded state, causing the proteolytic degradation to be much faster than the knotted protein. This finding also suggested that the knotted topology of UCHL1 likely plays a role to impede unfolding and degradation by ClpXP. This observation is consistent with a computational study of 5_2_ knotted polymers. These knots have high topological friction that sterically hinders translocation across a narrow pore in a process that resembles the translocation process made by ClpXP^48^. Furthermore, the mechanostability of a given protein depends on the functional requirements associated with malleability. On the one hand, the proteasome-associated UCHL5 is the least malleable among the four UCHs because it may need to withstand the pulling by the proteasome through competing the same ubiquitinated substrate. On the other hand, BAP1 is the most malleable, potentially because (partial) unfolding is required for it to be shuttled between the cytosol and nucleus through the nuclear pore complex. In light of the rapid advancements of cryoelectron microscopy in single particle reconstruction of heterogeneous structural ensembles of supramolecular assemblies, the superior mechanostability and slow turnover rates of the UCHs afford an opportunity for future structural investigation into how AAA+ proteasomes recognize, unfold and digest their substrates.

## Materials and Methods

### Cloning, protein production and purification

ClpX (ClpX ΔN-ter), ClpP, GFP_ssrA_ plasmid constructs were a kind gift of Dr. Robert T. Sauer (MIT, Cambridge, USA). All the UCH variants were cloned into a modified pET21a vector with a linker (HGMDELYK) connected to the ssrA tag (AANDENYALAA) at the C-terminus and a His-tag and TEV protease cleavage site at the N-terminus. These included: UCHL1, UCHL1_I93M_, UCHL1_∆11_, UCHL3, UCHL5 (residues 1–240), and BAP1 (residues1–238).

Over expression of all the above proteins was achieved in *E. coli* BL21 host cell harboring the gene of interest. Cells were grown at 37 °C in LB medium supplemented with 100 µg/mL ampicillin until the O.D._600_ reached 0.6–0.8. Protein over expression was induced by addition of 0.5 mM IPTG followed by 16 hrs of incubation at 16 °C. The cells were harvested by centrifuge at 6000 rpm for 20 min. The bacterial pellets were re-suspended with buffer-A (50 mM Tris-HCl (pH 8.0), 300 mM NaCl, 20 mM imidazole, 1% Triton X-100, 10 mM β-mercapto ethanol, 10% glycerol), lysed by sonication and clarified by centrifugation at 20,000 rpm for 30 minutes. The supernatant was loaded onto the Ni-NTA column, which was pre-equilibrated with buffer-A and washed extensively with buffer-B (50 mM Tris-HCl (pH 8.0), 300 mM NaCl, 20 mM imidazole). The target proteins were eluted with buffer-C (50 mM Tris-HCl pH 8.0, 300 mM NaCl, 250 mM imidazole), pure fractions of target proteins were collected and treated with Tobacco Etch Virus protease (1:50 TEV to target protein ratio) for 2 hours at room temperature and dialyzed the proteins against buffer-A to remove excess amount of imidazole. Protein samples were loaded onto a Ni-NTA column to remove TEV cleaved His-tag and the flow-through were collected for subsequent size-exclusion chromatography (SEC) by Superdex 75 16/60 column (GE Life science, USA). The purified proteins were further dialyzed against different buffers, based on the requirement of the experiments. A similar protocol was used to purify the GFP fused proteins, except that a Superdex 200 16/60 column (GE Life science, USA) was used during SEC. ClpX and ClpP proteins were expressed and purified following a previously described protocol^41,53^.

### Degradation assays

All the degradation assays of the ssrA-tagged substrate proteins were carried out in PD buffer (25 mM HEPES pH 7.6, 100 mM KCl, 10 mM MgCl_2_, 1 mM DTT, 10% glycerol (v/v)) supplemented with 5 mM ATP along with 16 mM creatine phosphate and 0.32 mg/mL creatine kinase for ATP regeneration. To a 100 µL total reaction volume 0.3 µM of ClpX_6_ and 0.9 µM ClpP_14_ were added and incubated for 2 minutes at 30 °C. To this reaction mixture, 3 µM of substrate protein was added and the aliquots (10 µL) were collected at various intervals. The reaction was quenched by addition of SDS loading dye (7 µL) and samples were flash frozen and stored at −30 °C. All the samples collected at various time points were loaded and separated on 12% SDS PAGE. The amount of intact substrate protein was quantified by ImageJ software.

Fluorescence-based degradation assays were performed in the same way as described above. The change in the fluorescence was measured by exciting the sample at 467 nm and recording the emission at 511 nm during the degradation reaction. For the Michaelis-Menten analyses, the GFP-fused substrate concentrations were set between 0.05 and 20 µM with a constant ClpXP concentration: 0.3 µM ClpX_6_ and 0.9 µM ClpP_14_. GFP fluorescence was monitored using an Infinite M1000PRO plate reader (TECAN, Switzerland) immediately after the reaction mixtures were prepared using a multichannel pipette in 96-well microplates. The experiments were carried at 30 °C.

### Chemical stability monitored by intrinsic fluorescence

Equilibrium unfolding data of the protein samples were acquired by chemical denaturation in guanidine hydrochloride gradient buffer condition. The intrinsic fluorescence of the substrate proteins was monitored by exciting the samples at 280 nm and recording the emission from 300 nm to 500 nm with an interval of 2 nm in Infinite M1000PRO plate reader (TECAN, Switzerland) at 25 °C. Denaturant induced equilibrium unfolding was studied in gradient concentrations of 0–6 M of guanidine hydrochloride in the PD buffer. A total of 24 data points were chosen, where the protein and buffer were mixed in 9:1 ratio that resulted in the 2 µM of protein final concentration. The samples were incubated for 16 hours at 25 °C before the intrinsic fluorescence measurements were collected.

The data was subjected to singular value decomposition (SVD) analysis using MATLAB (MATLAB and statistical Toolbox release 2012b, The MathWorks, USA) as described previously^22–29^. The numbers of the significant components were evaluated from the normalized correlation coefficients of individual singular values, with a minimum threshold of 0.8. All the equilibrium data were subjected to two-state or three-state equilibrium unfolding model as described previously^26^. The SVD components were fit globally to extract the free energy of unfolding (ΔG_u_) and the associated *m*-value and [D]_50%_ values.

### Thermal stabilities monitored by DSC

Differential scanning calorimetry (DSC) experiments were performed using Malvern MicroCal VP-Capillary DSC system (Malvern, UK). The substrate proteins were dialyzed against 25 mM HEPES pH 7.6, 100 mM KCl, 10 mM MgCl_2_, 10% glycerol (v/v) and 1 mM TCEP. 10 µM of sample was used to perform the experiment. The samples were heated from 10 to 90 °C at a rate of 200 °C h^−1^ under a pressure of 60 psi. For each experiment, a progress baseline was subtracted to remove ΔCp effect. The data was fit to independent non-two-state transitions model using the Microcal VP-capillary DSC analysis software.

### Folding kinetics monitored by stopped flow fluorescence measurements

The folding kinetics of the UCHs was monitored using a stopped-flow spectrometer in a fluorescence detection mode (SX18 stopped-flow spectrometer, Applied Photophysics, UK) as described previously^25,26,28,29^. 20 *µ*M of the protein was buffered in 20 mM Tris-HCl (pH 7.6), 100 mM NaCl with urea for unfolding measurements. Changes in total fluorescence were monitored using an excitation wavelength of 280 nm with a 320 nm cut-off filter. All experiments were carried out at 25 °C. 20 *µ*M of the protein was buffered in 20 mM Tris-HCl (pH 7.6), 100 mM NaCl with urea for refolding or without urea for unfolding measurements. The folding reactions were triggered by rapidly mixing the folding or unfolding buffer with protein solution at asymmetric mixing ratio of 9:1. After the 10-fold dilution, the final protein concentration was 2 *µ*M. For all kinetic analyses, the observed reaction rates were extracted by fitting the kinetic traces to a single, double, or triple exponential function with an offset using the software package GraphPad Prism (GraphPad Software, USA). The choice of model, *i.e*., number of kinetic phases, was decided using the F-test statistics.

The observed reaction rates in the stopped-flow fluorescence measurements were fit to a simple two-state folding model to extract the associated kinetic and thermodynamic parameters. The observed reaction rates (*k*_obs_), with a linear refolding arm and a linear unfolding arm, were fit to the following equation:

$${k}_{obs}={k}_{f}^{{H}_{2}O}{e}^{-\frac{{m}_{f}[den]}{RT}}+{k}_{u}^{{H}_{2}O}{e}^{-\frac{{m}_{u}[den]}{RT}}$$

where ${k}_{f}^{{H}_{2}O}$ and ${k}_{u}^{{H}_{2}O}$ are the folding and unfolding rate in the absence of denaturant, *m*_f_ and *m*_u_ are the *m*-values associated with folding and unfolding respectively, *R* is the gas constant, and *T* is the sample temperature, which is set to 298 K (25 °C).

### Data availability

The data sets regenerated during and/or analysed during the current study are available from the corresponding author on reasonable request.

## Electronic supplementary material

Supporting information
