# Supplementary material for: Topologically knotted deubiquitinases exhibit unprecedented mechanostability to withstand the proteolysis by an AAA+ protease
Source: Sci Rep. 2018 May 4;8:7076. doi: 10.1038/s41598-018-25470-0 (PMC5935755; doi:10.1038/s41598-018-25470-0)
Supplement: Supplementary file 1 — Supporting information [file 41598_2018_25470_MOESM1_ESM.pdf]

Supporting information

Topologically knotted deubiquitinases exhibit unprecedented mechanostability to withstand the proteolysis by an AAA+ protease

Manoj Kumar Sriramoju[1], Yen Chen[1], Yun-Tzai Cloud Lee,[1,2]
and Shang-Te Danny Hsu[1,2]*

1. Institute of Biological Chemistry, Academia Sinica, Taipei 11529, Taiwan
2. Institute of Biochemical Sciences, National Taiwan University, Taipei 106, Taiwan

* corresponding author: sthsu@gate.sinica.edu.tw

Table S1. Thermodynamics parameters of GdnHCl-induced equilibrium unfolding of ssrA-UCHs.

Protein		*m* (kcal mol^{-1} M^{-1})	ΔG (kcal mol^{-1})	$[D]_{50\%}$ (M)
UCHL1	N-I	4.09±0.40	2.79±0.35	0.68±0.02
	I-D	2.54±0.26	4.24±0.29	1.70±0.03
$UCHL1_{I93M}$	N-I	6.61±0.66	3.82±0.37	0.58±0.01
	I-D	2.14±0.16	3.76±0.19	1.62±0.03
$UCHL1_{\Delta 11}$		1.71±0.09	1.77±0.10	1.04±0.02
UCHL3		2.32±0.34	5.47±0.79	2.36±0.04
UCHL5		2.45±0.25	3.51±0.37	1.43±0.03
BAP1		1.78±0.12	3.63±0.24	2.04±0.02
GFP		2.76±0.27	8.31±0.81	3.01±0.02

Table S2. Melting temperatures and corresponding enthalpies of ssrA-UCHs determined by differential scanning calorimetry (DSC).

Protein	$T_{m,1}$ (°C)	$T_{m,2}$ (°C)	ΔH_1 (kcal M^{-1})	ΔH_2 (kcal M^{-1})
UCHL1	55.2±0.01	-	114.0±0.2	-
$UCHL1_{I93M}$	51.4±0.01	-	136.0±0.3	-
$UCHL1_{\Delta 11}$	45.7±0.04	-	28.4±0.3	-
UCHL3	58.5±0.02	-	86.7±0.4	-
UCHL5	54.2±0.24	57.9±0.04	31.9±2.7	54.0±2.6
BAP1	46.9±0.21	50.1±0.04	51.1±3.7	42.2±3.6
GFP	80.1±0.42	82.9±0.07	114.2±19.3	115.9±18.7

Table S3. Folding kinetics parameters of UCHs derived from chevron plot analysis

Protein	$k_f^{H_2O}$ (s^{-1})	m_f (kcal mol^{-1} M^{-1})	$k_u^{H_2O}$ (s^{-1})	m_u (kcal mol^{-1} M^{-1})
UCHL1	0.20±0.05	-1.21±0.07	$(7.0±0.6)*10^{-6}$	0.97±0.01
$UCHL1_{I93M}$	0.10±0.10	-1.28±0.37	$(2.2±0.5)*10^{-5}$	1.11±0.03
$UCHL1_{\Delta 11}$	N.D.	N.D.	$(2.1±0.4)*10^{-4}$	0.21±0.02
UCHL3	49±20	-1.28±0.10	$(2.6±0.9)*10^{-4}$	0.82±0.03
UCHL5	0.14±0.03	-1.09±0.07	$(8.1±7.6)*10^{-9}$	1.88±0.12
BAP1	N.D	N.D	$(1.2±0.9)*10^{-4}$	0.35±0.08

Table S4. Physico-chemical properties of ClpXP substrates compiled from the literature.

Proteins	K_M (μM)	$k_u^{H_2O}$ (min^{-1})	V_{max} (min^{-1} [ClpX$_6$]$^{-1}$)	ΔG_u[a] (kcal mol^{-1})	T_m (°C)	Ref.
Arc	1.5±0.1	8.4	1.8±0.1	1.3	62	1,2
PL8-Arc	1.0±0.2	0.12	1.3±0.1	2.2	74	1,2
FA10-Arc	1.2±0.1	184	2.1±0.1	-0.4	48	1,3
IV37-Arc	1.1±0.1	44	2.3±0.1	0.2	52	1,4
NC11-Arc	1.0±0.2	4.8x10^{-6}	1.3±0.1	14.6	>100	1
GFP	1.95	6.4x10^{-8}	0.9			5
I27 ssrA	1.4	0.026	0.25	6.4	71.3	1,6
V4A I27	1.1	0.17	0.36	4.4		1
Y9P I27	1.6	0.13	1.5	4.5	64.3	1,6
V11P I27	2.9	19	2.9	3.5		1
V13P I27	2.3	32	3.1	2.9		1
V15P I27	1.5	2.3	0.85	4.6		1
H6-RNaseH	0.9		3.7	12.0	82	7
H6-L78D	1.3		3.1	5.1	61	7
H6-L112D	0.9		4.4	3.1	52	7
RNaseH wt	0.9		4.2		82	7
^{SF}GFP	0.36	1.6x10^{-9}	0.39	6.9		8
CP7-^{SF}GFP	0.79	4.2x10^{-7}	1.02	4.4		8
Cp6-^{SF}GFP	0.33	2.9x10^{-9}	1.48	4.9		8
GFP	1.3	5.9x10^{-7}	1.19	4.6		8

a. The free energies of unfolding ΔG_u were all derived from GdnHCl-induced equilibrium unfolding.

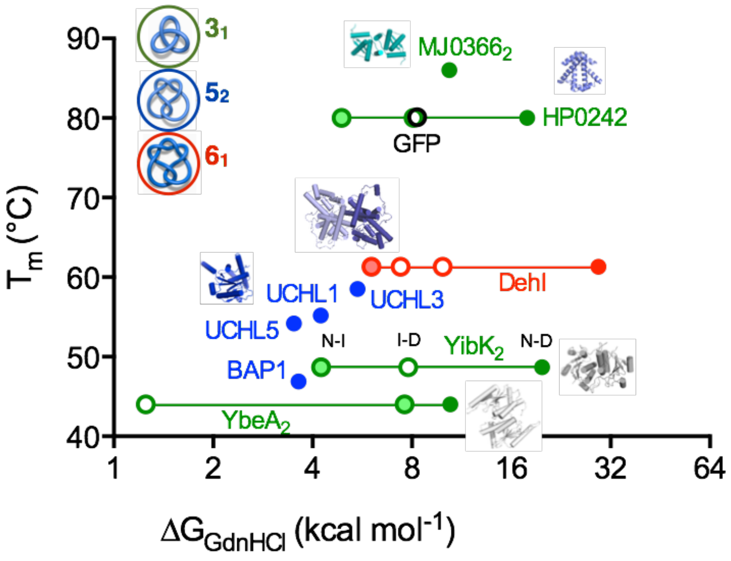

Figure S1. Correlation between chemical stability and thermal stability of knotted proteins that have been investigated so far. The data points corresponding to 3_1, 5_2 and 6_1 knotted proteins are shown in green, blue and red, respectively. The data point of GFP is shown in open black circle. The chemical denaturation of most knotted proteins exhibit well-defined folding intermediates. Therefore the free energy of unfolding ΔG_{GdnHCl} corresponding native-to-intermediate (N-I), intermediate to denatured (I-D), and native to denatured (N-D) states are shown in lightly filled, open and solid circles, respectively. For YibK, YbeA and MJ0366, the contributions of dimerization in chemical denaturation are considered during the calculation of the overall ΔG_{GdnHCl} of N-D transitions. To illustrate the relative sizes of the knotted proteins, the cartoon representations of the individual knotted proteins are shown in the same scale next to the data points.

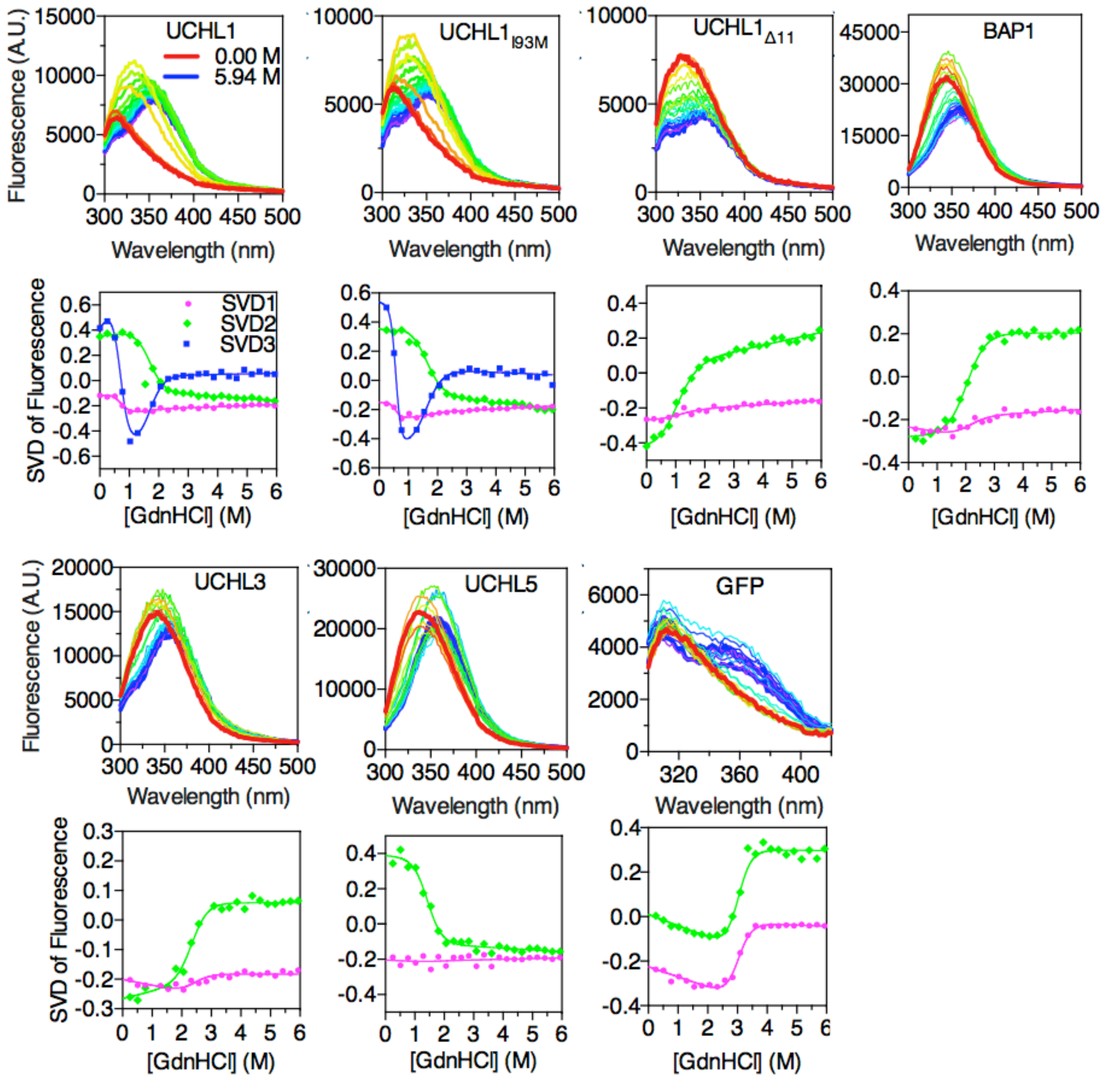

Figure S2. GdnHCl-induced equilibrium unfolding of ssrA-UCHs and ssrA-GFP monitored by intrinsic tryptophan fluorescence. The intrinsic fluorescence spectra were collected by excitation at 280 nm. The fluorescence spectra are color-ramped from red and blue on linearly incremented GdnHCl concentrations from 0 to 5.94 M with 24 titration points in total. The resulting spectra were subject to SVD analyses as described previously (see Material and Methods), and global-fit to a two- or three-state folding model as shown in solid lines.

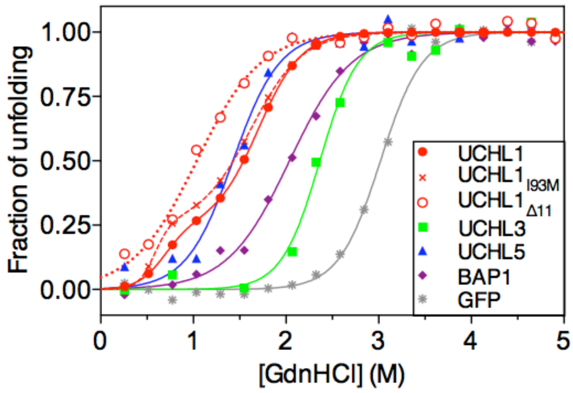

Figure S3. Normalized fraction of unfolding of ssrA-UCHs and ssrA-GFP derived from Figure S1. For UCHL1 and UCHL1$_{I93M}$, a three-state model was used to fit the equilibrium unfolding data. The relative intermediate populations were arbitrarily set to 0.5 to illustrate the three-state unfolding process.

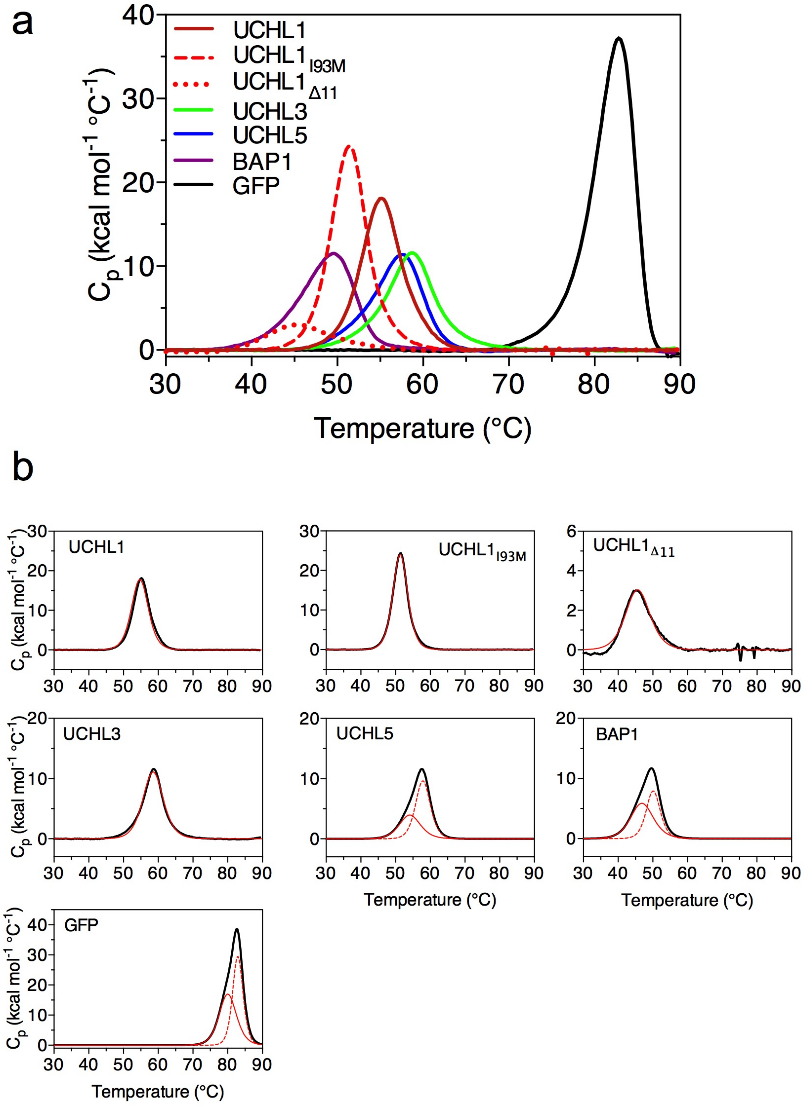

Figure S4. Thermal unfolding of ssrA-UCHs and ssrA-GFP monitored by DSC. **A.** Comparison of the DSC profiles of all ssrA-tagged substrate in the same scale. **B.** Raw data (black lines) and fitting results (solid and dashed red lines for the first and second transitions, respectively) of individual substrates.

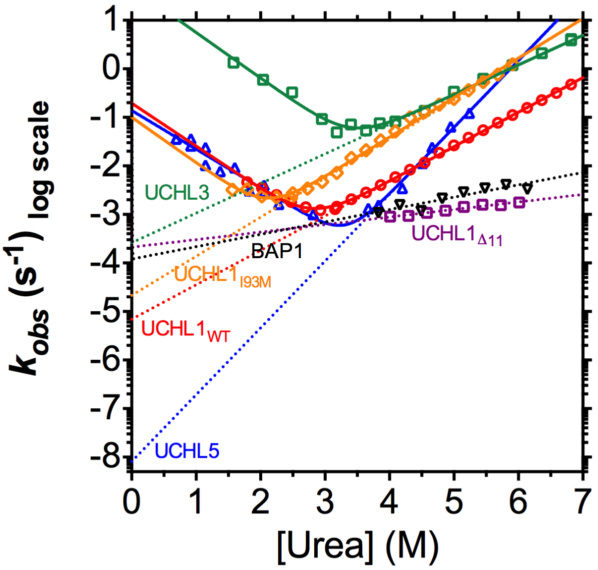

Figure S5. Chevron plot of UCHs. The slowest unfolding arms of all UCHs (without ssrA tag) were extrapolated to 0 M urea concentration to estimate the intrinsic unfolding rates, $k_u^{H_2O}$. For BAP1 (inverted black triangles) and UCHL1$_{\Delta 11}$ (open purple squares), only unfolding rates were determined. The chevron plots of UCHL3 (open green squares) and UCHL5 (open blue triangles) were taken from previously reported study [9], while the chevron plots of UCHL1 (open red circles) and UCHL1$_{I93M}$ (open orange diamonds) were newly collected in this study.

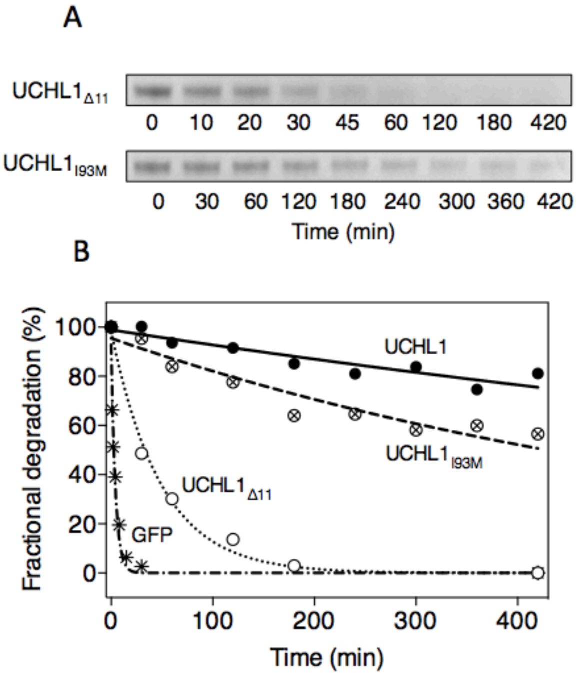

Figure S6. ClpXP-mediated proteolysis of ssrA-tagged $UCHL1_{I93M}$ and $UCHL1_{\Delta 11}$ monitored by SDS-PAGE. (A) SDS-PAGE images of individual substrates as indicated on the left of each panel. Aliquots were taken at specific time points as indicated below. (B) Quantitative image analyses of the results shown in (A) normalized with respect to the initial time point. The results were fit to a single exponential decay function to deduce the apparent life times of individual substrates.

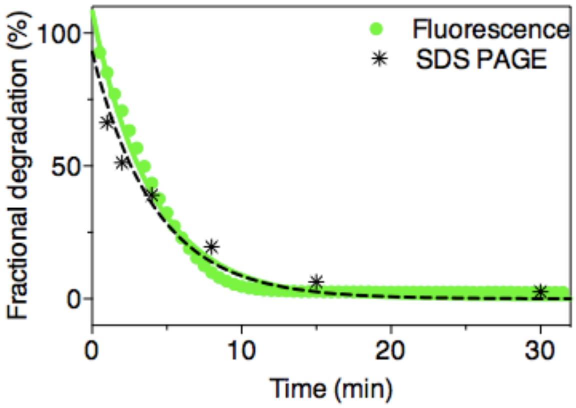

Figure S7. Comparison of ClpXP-mediate proteolysis of ssrA-tagged GFP monitored by SDS-PAGE and GFP fluorescence. The observed changes were fit to a single exponential decay function as indicated by black dashed line and solid green line, respectively.

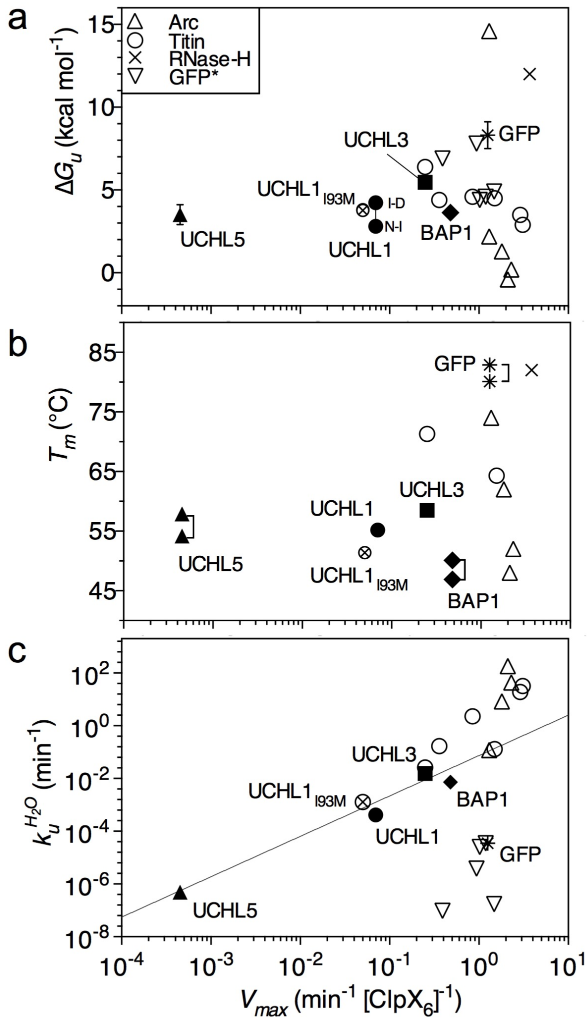

Figure S8. Correlation plots of ClpXP-mediated proteolysis rates, $V_{\max}$, with respect to (A) free energy of unfolding derived from equilibrium unfolding by GdnHCl, ΔG_u, (B) melting temperature, T_m, and (C) intrinsic unfolding rate $k_u^{H_2O}$. The parameters associated with Arc (open triangles), titin I27 (open circles), RNase-H (cross) and GFP (inverted open triangles) were compiled from the literature (*cf.* **Table S4**).[1-8] The non-linear regression of the correlation between $V_{\max}$ and $k_u^{H_2O}$ for UCHs are shown in solid line in (C). For UCHL1 and $UCHL1_{I93M}$, two GdnHCl-induced transitions were observed, corresponding to N-I and I-D transitions, which are indicated for UCHL1, but for $UCHL1_{I93M}$ the two values are very similar so only one symbol is visible in (A). For UCHL5 and BAP1, two transition temperatures were observed in DSC, which are linked by brackets in (B).

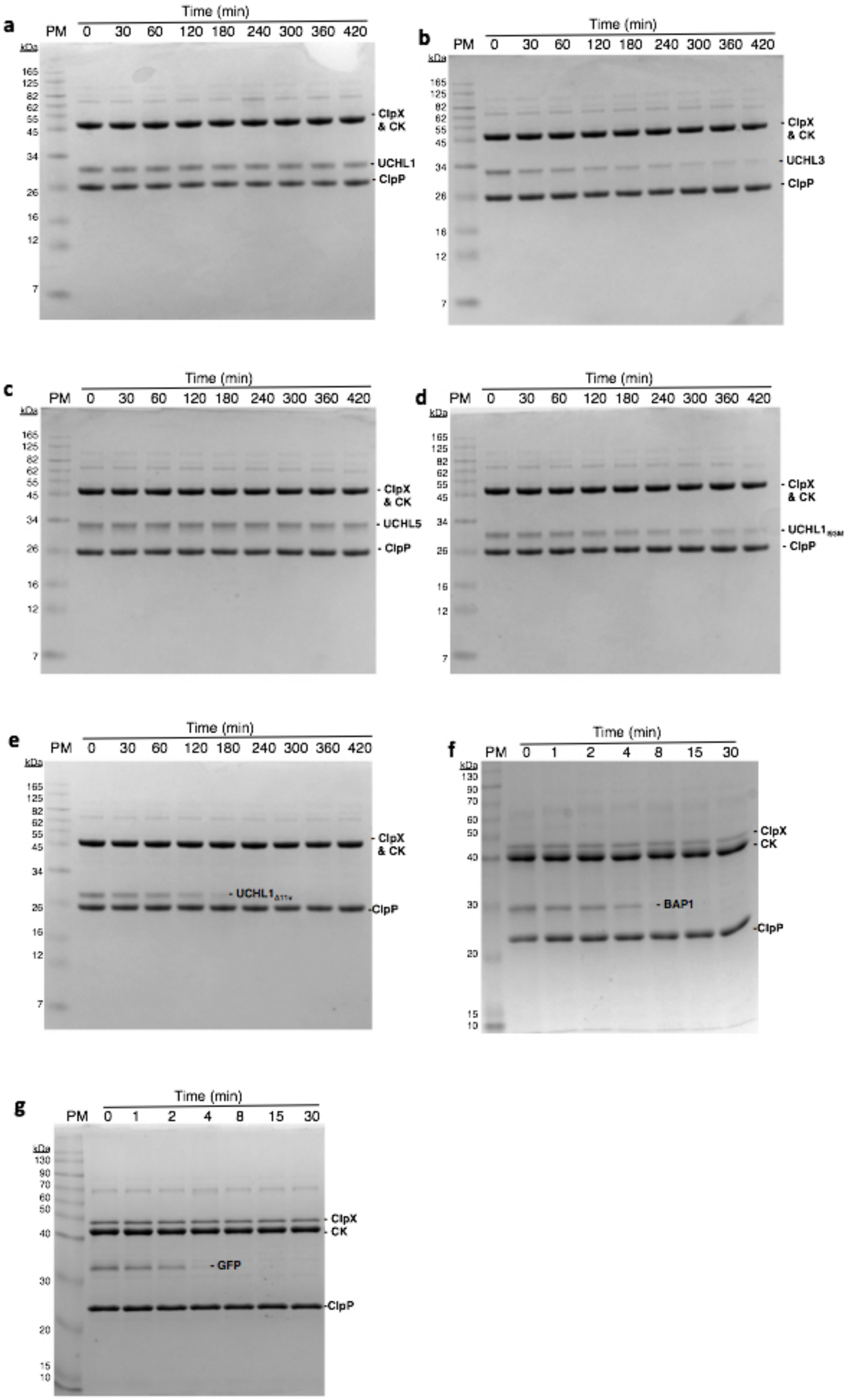

Figure S9. Original SDS-PAGE images of ClpXP-mediated degradation assays of ssrA-tagged UCHL1 (a), UCHL3 (b), UCHL5 (c), UCHL1$_{I93M}$ (d), UCHL1$_{\Delta 11}$ (e), BAP1 (f), GFP (g). The protein samples were separated on 12% SDS PAGE Gels

References

1. Kenniston, J.A., Baker, T.A., Fernandez, J.M. & Sauer, R.T. Linkage between atp consumption and mechanical unfolding during the protein processing reactions of an aaa+ degradation machine. *Cell* **114**, 511-20 (2003).
2. Schildbach, J.F., Milla, M.E., Jeffrey, P.D., Raumann, B.E. & Sauer, R.T. Crystal structure, folding, and operator binding of the hyperstable arc repressor mutant pl8. *Biochemistry* **34**, 1405-12 (1995).
3. Milla, M.E., Brown, B.M., Waldburger, C.D. & Sauer, R.T. P22 arc repressor: Transition state properties inferred from mutational effects on the rates of protein unfolding and refolding. *Biochemistry* **34**, 13914-9 (1995).
4. Milla, M.E. & Sauer, R.T. Critical side-chain interactions at a subunit interface in the arc repressor dimer. *Biochemistry* **34**, 3344-51 (1995).
5. Kim, Y.I., Burton, R.E., Burton, B.M., Sauer, R.T. & Baker, T.A. Dynamics of substrate denaturation and translocation by the clpxp degradation machine. *Mol Cell* **5**, 639-48 (2000).
6. Oroz, J. et al. The y9p variant of the titin i27 module: Structural determinants of its revisited nanomechanics. *Structure* **24**, 606-616 (2016).
7. Kenniston, J.A., Burton, R.E., Siddiqui, S.M., Baker, T.A. & Sauer, R.T. Effects of local protein stability and the geometric position of the substrate degradation tag on the efficiency of clpxp denaturation and degradation. *J Struct Biol* **146**, 130-40 (2004).
8. Nager, A.R., Baker, T.A. & Sauer, R.T. Stepwise unfolding of a beta barrel protein by the aaa+ clpxp protease. *J Mol Biol* **413**, 4-16 (2011).
9. Lee, Y.T.C. et al. Entropic stabilization of a deubiquitinase provides conformational plasticity and slow unfolding kinetics beneficial for functioning on the proteasome. *Sci Rep* **4**, 45174 (2017).
